# Auxin Response Factors (ARFs) are potential mediators of auxin action in tomato response to biotic and abiotic stress (*Solanum lycopersicum*)

**DOI:** 10.1371/journal.pone.0193517

**Published:** 2018-02-28

**Authors:** Sarah Bouzroud, Sandra Gouiaa, Nan Hu, Anne Bernadac, Isabelle Mila, Najib Bendaou, AbdelAziz Smouni, Mondher Bouzayen, Mohamed Zouine

**Affiliations:** 1 GBF, Université de Toulouse, INRA, Castanet-Tolosan, France; 2 Laboratoire de physiologie et biotechnologie Végétales, Centre de biotechnologie végétale et microbienne biodiversité et environnement, Faculté des Sciences, Université Mohammed V de Rabat, Rabat, Maroc; National Taiwan University, TAIWAN

## Abstract

Survival biomass production and crop yield are heavily constrained by a wide range of environmental stresses. Several phytohormones among which abscisic acid (ABA), ethylene and salicylic acid (SA) are known to mediate plant responses to these stresses. By contrast, the role of the plant hormone auxin in stress responses remains so far poorly studied. Auxin controls many aspects of plant growth and development, and Auxin Response Factors play a key role in the transcriptional activation or repression of auxin-responsive genes through direct binding to their promoters. As a mean to gain more insight on auxin involvement in a set of biotic and abiotic stress responses in tomato, the present study uncovers the expression pattern of *SlARF* genes in tomato plants subjected to biotic and abiotic stresses. *In silico* mining of the RNAseq data available through the public TomExpress web platform, identified several *SlARF*s as responsive to various pathogen infections induced by bacteria and viruses. Accordingly, sequence analysis revealed that 5’ regulatory regions of these SlARFs are enriched in biotic and abiotic stress-responsive cis-elements. Moreover, quantitative qPCR expression analysis revealed that many SlARFs were differentially expressed in tomato leaves and roots under salt, drought and flooding stress conditions. Further pointing to the putative role of SlARFs in stress responses, quantitative qPCR expression studies identified some miRNA precursors as potentially involved in the regulation of their SlARF target genes in roots exposed to salt and drought stresses. These data suggest an active regulation of SlARFs at the post-transcriptional level under stress conditions. Based on the substantial change in the transcript accumulation of several SlARF genes, the data presented in this work strongly support the involvement of auxin in stress responses thus enabling to identify a set of candidate SlARFs as potential mediators of biotic and abiotic stress responses.

## Introduction

Tomato (*Solanum lycopersicum*) is one of the major crops with significant economic and scientific interests and as in the case of other crop plants, environmental stresses negatively affect tomato growth, productivity and quality [1]. Developing new tomato cultivars with enhanced tolerance to abiotic and biotic stress would have a significant impact on global food production in many regions. Plant responses to environmental stresses are extremely complex and involve changes at the transcriptome, cellular and physiological levels in order to prevent damage and ensure survival [2]. Better understanding of the molecular mechanisms by which plants adapt to constantly changing environmental conditions is a topic of prime interest in the context of global climate warming. However, our knowledge of the actors and pathways underlying plant tolerance or susceptibility to environmental stresses remains scarce. Several phytohormones are known for their role in the response to abiotic and biotic stresses [1]. Abiotic stress responses are largely controlled by the hormone ABA while defense against different biotic constraints is specified by antagonism between the salicylic acid (SA) and jasmonic acid (JA) or ethylene signaling pathways [3,4]. This creates a complex network of interacting pathways and hormones cross-talk at different levels [5].

Even though auxin is known to control a wide range of plant developmental processes, strikingly, its putative role in modulating plant growth under stress responses remains poorly understood. Auxin is involved in different aspects of plant development throughout the plant life cycle, including apical dominance, tropic responses, vascular development, organ patterning, flower and fruit development [6–12]. Significant progress has been made towards understanding the mechanisms by which this hormone impacts plant growth and development. By contrast, our knowledge about auxin implication in biotic and abiotic stress responses remains quite limited [13]. Recent studies including expression profiling suggested that auxin might act as a regulator of plant responses to environmental stresses [11,14–18].

Auxin regulates the cell-specific transcription of auxin response genes via three types of transcriptional regulators, Auxin/Indole-Acetic Acid (Aux/IAAs), Auxin Response Factors (ARFs) and TOPLESS proteins (TPS) [19–21]. In *Arabidopsis*, 23 ARFs were identified based on the presence of a conserved N-terminal DNA-binding domain (DBD), a variable central transcriptional regulatory region which can function as activator or a repressor domain, and a carboxy-terminal dimerization domain (CTD) that contributes to the formation of either ARF/ARF homo- and hetero-dimers or ARF/Aux/IAA hetero-dimers [21–23]. The DBD enables ARFs to specifically bind the conserved Auxin Response Element (AuxRE, 5’ TGTCTC 3’) present in the promoters of various Auxin-regulated genes and in this way to control gene transcription associated with plant responses to auxin [23–26].

ARF proteins orchestrate several biological and physiological processes such as embryogenesis, leaf expansion and senescence, lateral root development and fruit development by regulating the expression of auxin response genes [27–30]. The ARF gene family has been identified and well characterized in many crop species, such as Arabidopsis (*Arabidopsis thaliana*) [31], Maize (*Zea mays*) [32], Rice (*Oryza sativa*) [11,15], Poplar (*Populus trichocarpa*) [33], Tomato (*Solanum lycopersicum*) [21,34,35], Chinese cabbage (*Brassica rapa*) [36], Sorgho (*Sorghum bicolor*) [18], and Banana (*Musa acuminata*) [37]. In recent years, small RNA, known as miRNA, have been shown to substantially contribute to the regulation of plant development, physiology and stress responses [38]. To date, 872 miRNAs, belonging to 42 families, have been identified in 71 plant species by genetic screening, direct cloning, computational strategies and EST analysis [38,39]. Noteworthy, among the first miRNAs to be identified and characterized, *miR160* has sequence complementarities with *ARF10*, *ARF16* and *ARF17* [21,40] and *miR167* potentially regulates *ARF6A*, *ARF6B* and *ARF8A* [21,41]. Therefore, *ARF* expression in tomato is likely to be regulated at the post-transcriptional level by a large family of miRNAs that are non-coding RNAs playing a critical role in regulating auxin-dependent gene expression at the post-transcriptional level [42,43].

To address the putative involvement of tomato *SlARF* genes in plant responses to stresses we performed in the present study *in silico* analyses of the 5’ regulatory regions of using PLACE web plateform (https://sogo.dna.affrc.go.jp/cgi-bin/sogo.cgi?lang=en) [44] and established their expression profiles under biotic stress conditions using TomExpress web platform [45]. We then, assessed the transcript accumulation of *SlARF* genes under salt, drought and flooding stresses using a qPCR experimental approach. We also examined the expression of miRNA precursor genes to check whether their expression is regulated under stress conditions and whether they are involved in post-transcriptional regulation of their target *ARF* genes under these conditions. Overall, the study provides guidance on the implication of auxin signaling pathway in plant responses to biotic and abiotic stresses and defines new targets towards engineering tomato plants better adapted to adverse environmental conditions.

## Materials and methods

### Plant materials

Tomato (*Solanum lycopersicum* cv Micro-Tom) plants of Wild type and transgenic DR5::GUS, pARF8A::GUS and pARF10::GUS lines generated in house (GBF laboratory) were used in this study.

### Histochemical analysis of GUS expression

Transgenic plants expressing pARF8A::GUS and pARF10A::GUS were generated by *A*. *tumefaciens*-mediated transformation according to Wang et al. (2005) [46]. For that, PCR was performed on the genomic DNA of tomato ‘Micro-Tom’ (10 ng.ml^–1^) using specific primers. The corresponding amplified fragment was cloned into the pMDC162 vector containing the GUS reporter gene using Gateway technology (Invitrogen). The cloned SlARF promoter was sequenced from both sides using vector primers in order to see whether the end of the promoter is matching with the beginning of the reporter gene. Sequence results were carried out using the Vector NTI (Invitrogen) and ContigExpress software by referring to ARF promoter sequences.

GUS assays were conducted on DR5::GUS, pARF8A::GUS and pARF10::GUS tomato lines. After being surface sterilized, seeds were cultivated in Petri dishes containing half strength Murashige & Skoog medium for 7 days in a growth chamber at 25°C with 16h light/ 8h dark cycle. One week plants were then grown hydroponically for two weeks in BD (BROUGHTON & DILLWORTH) liquid medium [47]. Three week-old plants were subjected to salt and drought treatment. Salt stress was performed by adding 250 mM of NaCl to the culture medium. After 24 hours of the salt stress application, plants were incubated in GUS solution. Drought stress was conducted by adding 15% of PEG 20000 to the liquid culture solution. Plants were collected after 5 days of stress application and were incubated in the GUS solution. For each stress condition, control plants were cultivated in BD liquid medium for the same period.

GUS staining was performed overnight at 37°C in 3 mM XGluc (5-bromo-4-chloro-3-indolyl-β-D-glucuronide (Duchefa Biochemie, Haarlem, The Netherlands), 0.1% (v/v) Triton X-100 Sigma, Steinhaim, Germany, 8 mM β-mercaptoethanol and 50 mM Na_2_HPO_4_/NaH_2_PO_4_ (pH 7.2), then followed by a destaining in EtOH.

### Plant growth and stress conditions

Wild type tomato seeds were sterilized for 10 min in 50% sodium hypochlorite, rinsed four times with sterile distilled water and sown in pots containing peat. Then they were incubated in a culture room with 16h light/ 8h dark photoperiod and 25± 2°C temperature. After 3 weeks, plants were subjected to salt, drought and flooding stresses. Salt stress was performed by watering daily the plants with 250mM of NaCl solution. Control plants were daily watered with distilled water. Leaves and roots samples were harvested after 2 and 24 hours of salt stress application. Drought stress was performed on three week-old plants by water holding for 48 hours and for 5 days. Watering continued normally throughout for control plants. Leave and root samples were collected after drought stress application. For flooding stress, three week-old plants were flooded with deionized water which was maintained at the level of cotyledonary node throughout the experiment. After 48 hours, the leaves and roots were harvested. Control plants were daily watered. Three biological replicates were done for each condition and three independent biological replicates were done for each experiment.

### RNA extraction

Total RNA was extracted from leaves and roots samples by using the Plant RNeasy extraction kit (RNeasy Plant Mini Kit, Qiagen, Valencia, CA, USA). To remove any residual genomic DNA, the RNA was treated with an RNase-Free DNase according to the manufacturer’s instruction (Ambion® DNA-*free*^TM^DNase). The concentration of RNA was accurately quantified by spectrophotometric measurement and 1μg of total RNA was separated on 2% agarose gel to monitor its integrity. DNase-treated RNA (2μg) was then reverse-transcribed in a total volume of 20μl using the Omniscript Reverse Transcription Kit (Qiagen).

### Real time PCR

The real-time quantification of cDNA corresponding to 2μg of total RNA was performed in the ABI PRISM 7900HT sequence detection system (Applied biosystems) using the QuantiTech SYBR Green RT-PCR kit (Qiagen). The Gene-specific primers used are listed in S1 Table. The reaction mixture (10μl) contained 2μg of total RNA, 1,2 μM of each primer and appropriate amounts of enzymes and fluorescent dyes as recommended by the manufacturer. Actin gene was used as reference. Real-Time PCR conditions were as follow: 50°C for 2 min, 95°C for 10 min, then 40 cycles of 95°C for 15 s and 60°C for 1 min, and finally one cycle at 95°C for 15 s and 60°C for 15 s. Three independent biological replicates and three technical replicates of each sample were used for real-time PCR analysis. For each data point, the C_T_ value was the average of C_T_ values obtained from the three biological replicates and three technical replicates.

Three stress related genes were used as markers in these experiments. *CI7* gene was used in both leaves and roots for salt and drought stresses [48,49]. For flooding, *ACO1* gene was used as a marker in leaves and *PDC* gene in roots [50].

### Tomato ARF gene expressions under biotic stress conditions

The expression pattern of 22 *SlARF* genes was analyzed by RNAseq technology in several studies and the results presented here were extracted from the TOMEXPRESS database (published online on October 2014) (S2 Table). TOMEXPRESS database includes 16 RNA-seq projects and 124 unique global conditions [45].

### Analysis of Cis-acting regulatory elements

Using database associated search tools, 2 kb of 5′ regulatory region of each *ARF* transcription factor gene in tomato was scanned for the presence of putative *cis*-acting regulatory elements and motifs registered in Plant PLACE database (https://sogo.dna.affrc.go.jp/cgi-bin/sogo.cgi?lang=en) (consulted in March 22^nd^, 2015) [44]. The *cis*-acting elements analyzed are listed in S3 Table.

### Statistical analysis

Data presented in this work are expressed as arithmetic means +/- SD of replicate plants within an experiment. The data shown are representative of a total of three independent biological replicates. The results were statistically analyzed using Student’s t test; Statistica soflware version 10 (Statsoft, Tulsa, USA). A p value of <0,05 was considered statistically significant. In all the figures presented, error bars indicate standard deviation.

## Results

### Analysis of SlARF promoter genes to search for stress response cis-acting regulatory elements

PLACE signal scan analysis revealed the presence of several *cis*-regulatory elements that are putatively associated with plant response to stress in the 5’ regulatory region (2kb upstream region from the translation start codon) of *SlARFs* (S1 Fig). The names of the identified *cis*-acting elements and their predicted functions are listed in S3 Table. Some of these elements (CCATBOX1, WBOXNTERF3, RAV1AAT, MYCCONSENSUSAT, GT1GMSCAM4, MYB2A; MYBCORE and MYBCONCENSUSAT) are common to the majority of the analyzed *SlARFs*. A high number of these conserved motifs is identified for *SlARF1*, *SlARF2A*, *SlARF4*, *SlARF6B*, *SlARF9A*, *SlARF16A* and *SlARF16B* whereas a low number are found for *SlARF10A* and *SlARF10B* genes.

Abiotic stress associated *cis*-acting regulatory elements were found for all *SlARF*s (S1 Fig). MYBCORE *cis*-acting element related to drought stress response [51] was detected in most of the 22 *SlARFs* except for *SlARF5*, *SlARF10A* and *SlARF24* while the MYB1AT [52] motif was only located in 13 of the 22 *SlARFs*. Noteworthy, MYCONSENSUSAT motif [53] was found in all the 22 *SlARFs* genes with varying occurrence. MYCATERD1 motif [52] was only found in *SlARF1*, *SlARF6A*, *SlARF7B*, *SlARF8A*, *SlARF8B*, *SlARF9B*, *SlARF16A* and *SlARF24* while the presence of the other MYC binding site “MYCATRD22” [51] was specific to *SlARF4*, *SlARF5*, *SlARF7A*, *SlARF7B*, *SlARF8B*, *SlARF16A* and *SlARF16B*. Screening for motifs involved in temperature variation showed that with the exception of *SlARF8A* and *SlARF18*, all other *SlARFs* promoter regions were enriched with the two *cis*-acting elements RAV1AAT and CCAATBOX1 boxes [54,55]. *Cis-*acting element associated with biotic stress response such as WBOXNTCHN48, WBOXNTERF3 and GT1GMSCAM4 were located in most *SlARF* promoters with differences in their occurrence. The WBOXNTERF3 motif, involved in wounding [56], was found in the SlARFs gene promoters except for *SlARF3*, *SlARF7A* and *SlARF9B* while the elicitor binding motif WBOXNTCHN48 [57] was only detected in *SlARF2A*, *SlARF5* and *SlARF6B*. Finally, the GT1GMSCAM4 motif [58] was present in all the *SlARFs* promoter regions.

### Tomato ARF responsiveness to biotic stress

**Fig 1:**
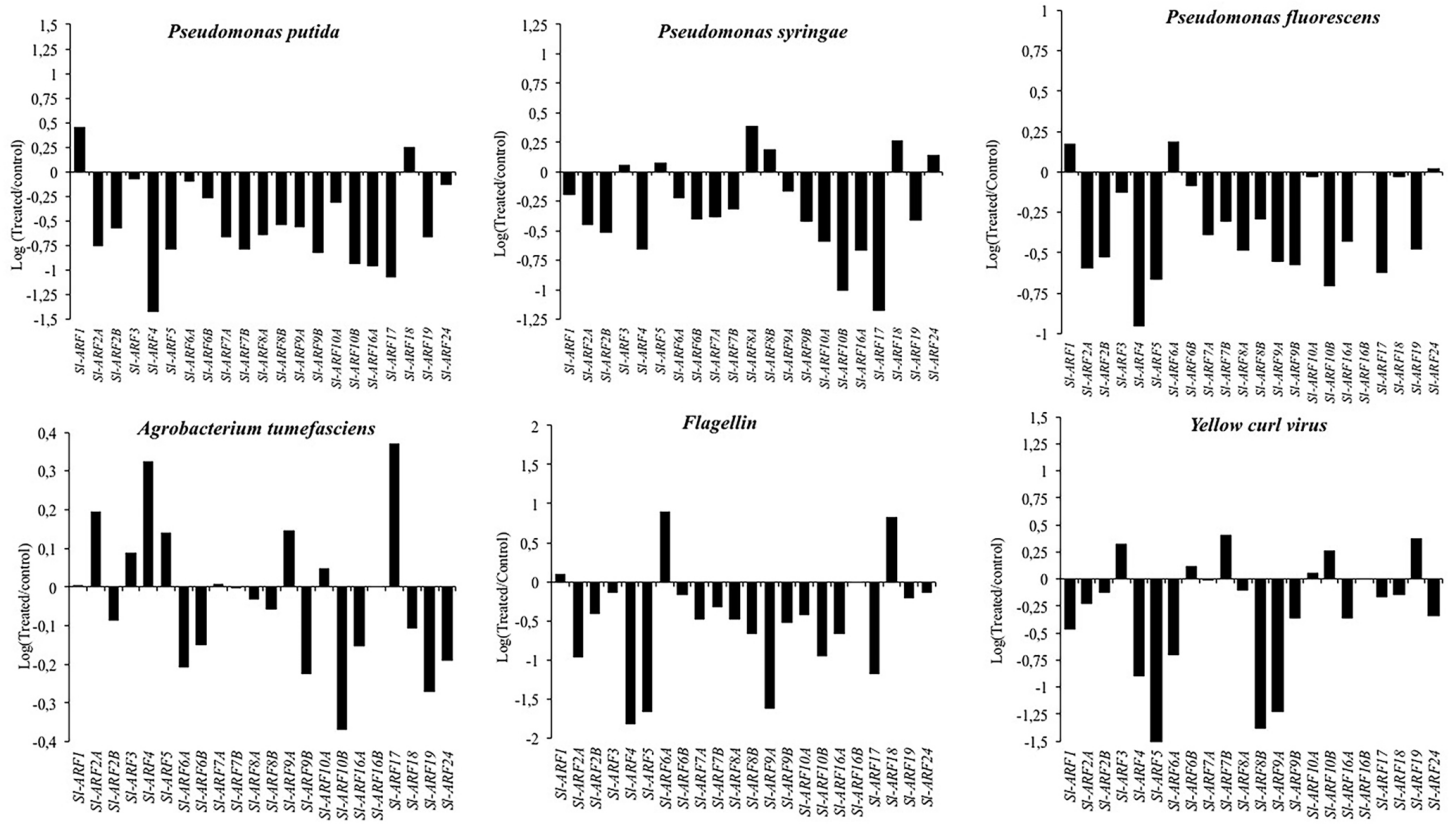
*SlARF*s gene expression in tomato plant leaves exposed to various biotic stresses: Flagellin, *Pseudomonas putida*, *Pseudomonas syringae*, *Pseudomonas fluorescens*, *Agrobacterium tumefaciens* and *Yellow curl virus*. All the data presented here were extracted from TOMPEXPRESS database (http://gbf.toulouse.inra.fr/tomexpress).

We mined the public tomato RNA-Seq web platform TomExpress [59] to examine the expression profile of tomato *SlARF* genes upon biotic stress. Among the expression data available in the TomExpress platform, we focused on those related to flagellin, bacteria (*Pseudomonas putida*, *Pseudomonas fluorescens*, *Pseudomonas syringae* DC3000 and *Agrobacterium tumefaciens*) and Yellow curl leave virus YCV infection (Fig 1). When tomato leaves were exposed six hours to *Pseudomonas* strains (*Pseudomonas putida*, *Pseudomonas fluorescens* or *Pseudomonas syringae* DC3000), most *SlARF* genes were down-regulated.

Flagellin is considered as the main building unit of the bacterial motility organ and constitutes an important microbial pattern for virulence through its use by plants to recognize bacterial pathogens. When tomato leaves are exposed six hours to flagellin [60], the expression of almost all members of the *SlARF* family is repressed except for *SlARF6A* and *SlARF18* that showed a twofold induction.

In response to *P*. *putida*, the expression of *SlARFs* was also repressed except for *SlARF1* and *SlARF18*. Likewise, upon *P*. *syringae* infection the expression of the overwhelming majority of *SlARF* genes was downregulated although *SlARF8A* was induced. *P*. *fluorescens* infection also resulted in down-regulation of most ARF genes, regulation that is highly marked for *SlARF4*. Noteworthy, none of the 22 *SlARFs* showed a significant change in their expression at the transcription level upon *Agrobacterium tumefaciens* infection.

Tomato yellow leaf curl virus (TYLCV) designates a complex of geminiviruses infecting tomato cultures worldwide. This virus is transmitted by a single insect species, the whitefly *Bemisia tabaci*. In response to TYLCV, the expression of *SlARF4*, *SlARF5*, *SlARF6A*, *SlARF8B* and *SlARF9A* genes was downregulated in leaf tissues whereas the expression of the remaining *SlARFs* did not exhibit significant change. Overall, down-regulation seems to be clearly the main trend for *SlARF* genes upon bacterial infection.

### Tomato ARF responsiveness upon abiotic stress

#### Distribution of auxin response activated upon abiotic stress

**Fig 2:**
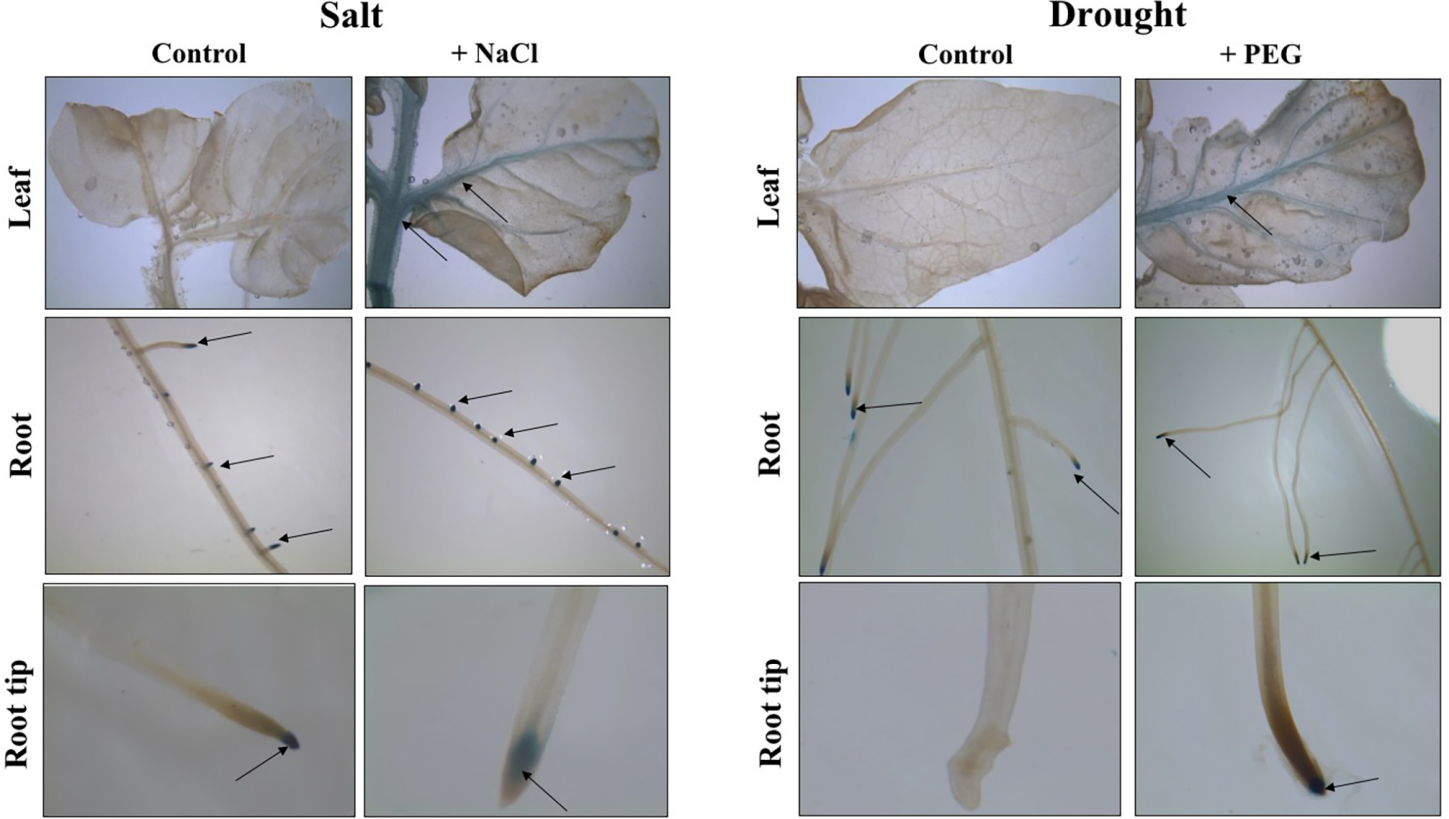
GUS activity in DR5::GUS tomato lines in salt or drought stress conditions. Salt and drought stresses were performed on three week-old tomato plants by adding 250 mM of NaCl or 15% PEG 20000 to the nutrient solution. Black arrows show the location of the GUS activity in the different tissues analyzed.

The extent of changes in auxin distribution and auxin signaling upon application of salt and drought stresses, were assessed using tomato lines expressing the GUS reported gene driven by the DR5 auxin-responsive promoter [61]. DR5::GUS tomato transgenic lines were subjected to salt and drought stress by adding 250 mM of NaCl and 15% PEG20000 to the nutrient solution, respectively (Fig 2). In leaf organs, GUS activity appears after 24 hours of salt stress and is localized in leaf veins and petioles while it is barely detectable in untreated plants. In salt stressed roots, the distribution of GUS expression is observed in lateral root primordia and in primary root tip. The control plants showed also a blue coloration in primary and lateral root tips. After 5 days of PEG treatment, a slight increase in GUS activity was detected in leaf veins and primary and lateral root tips while it scarcely appears in lateral root primordia of the untreated plants.

#### Expression profiling of SlARF genes in response to salt stress

**Fig 3:**
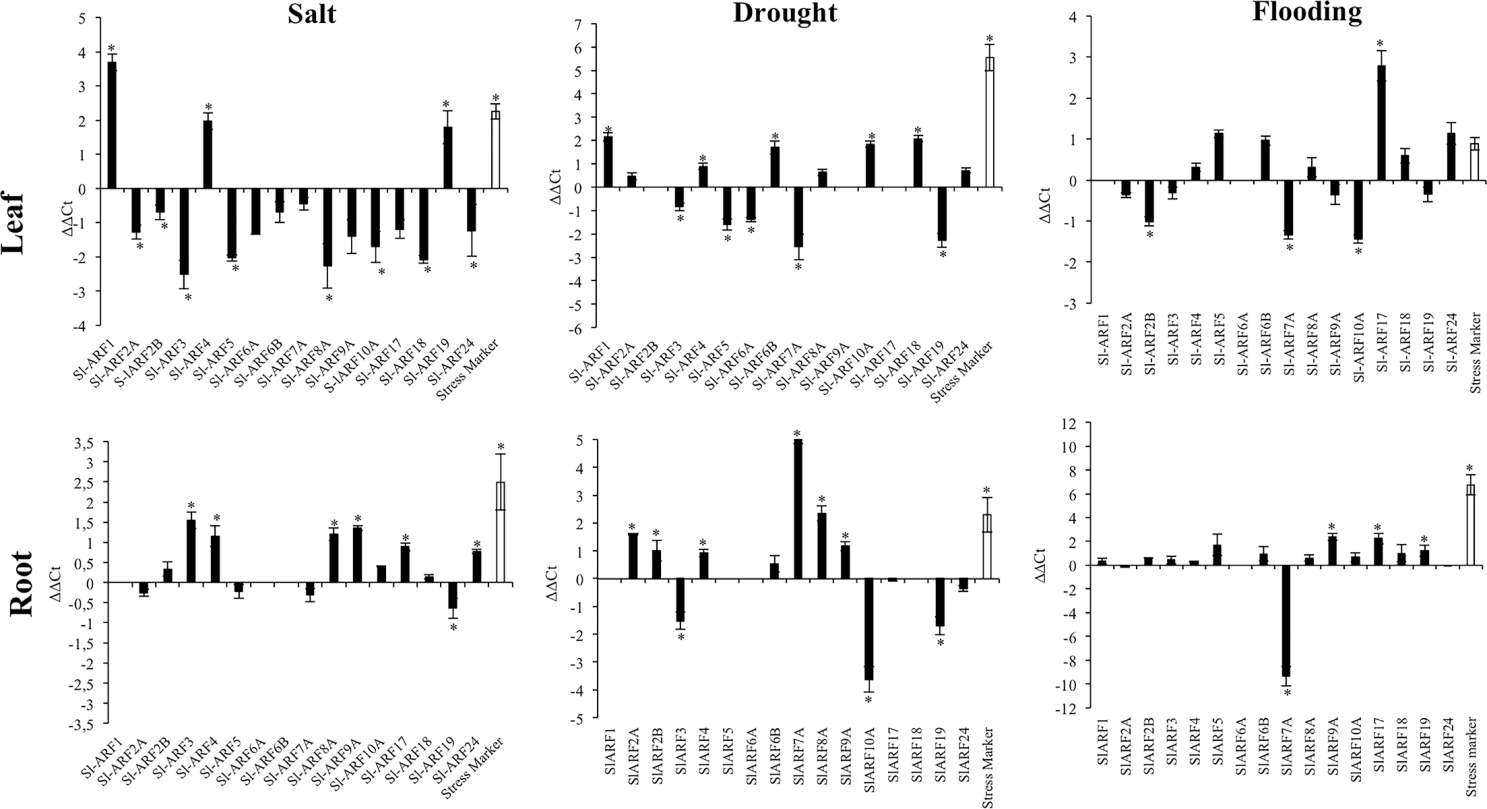
*SlARF*s gene expression under salt, drought and flooding conditions. Values are mean ± SD of three biological replicates. White bars represent the expression of stress marker: *CI7* for salt and drought stresses (Leaves & Roots), *ACO1* in flooded leaves and PDC1 in flooded roots. Stars (*) indicate the statistical significance (p<0,05) using Student’s t-test.

We analyzed the expression profiles of *SlARF* genes in tomato leaves and roots after 24 hours of salt treatment. The expression of *Cold Inducible 7* gene (*CI7)*, a known marker for salt stress [48], was significantly induced in both leaves and roots as expected, thus validating the efficiency of the applied stress in our condition. The data reveal that almost all *SlARF* genes were downregulated in leaves except *SlARF1*, *SlARF4* and *SlARF19* whose expression was significantly induced (Fig 3). Among the down-regulated genes, *SlARF3*, *SlARF5*, *SlARF8A* and *SlARF18* were highly repressed. In roots, 9 *SlARF* genes were up regulated among which *SlARF3*, *SlARF4*, *SlARF8A* and *SlARF9A* showed high induction (Fig 3). The expression of *SlARF2A*, *SlARF5* and *SlARF7A* were slightly repressed though not statistically significant while the expression of *SlARF19* was significantly repressed.

#### Expression profiling of SlARF genes in response to drought stress

The responsiveness of *SlARF* members under water deficit condition was investigated by qPCR after five days of drought stress application. The drought marker gene *C17*, unsurprisingly, showed the same pattern (high induction) in leaves and roots as previously reported by Kirch et *al*., (1997) [48]. In leaves, solely, *SlARF1*, *SlARF4*, *SlARF6B*, *SlARF10A* and *SlARF18* showed significant increase in expression, while the expression of *SlARF3*, *SlARF5*, *SlARF6A*, *SlARF7A* and *SlARF19* was significantly repressed (Fig 3).

In roots, the expression of *SlARF2A*, *SlARF2B*, *SlARF4*, *SlARF7A*, *SlARF8A* and *SlARF9A* was significantly increased upon drought stress while *SlARF3*, *SlARF10A*, *SlARF19* displayed significant down-regulation. Of particular note, *SlARF7A* with 32 fold induction displayed the most dramatic up-regulation compared to *CI7* control gene which showed only four times increase in the stressed roots (Fig 3).

#### Expression profiling of SlARF genes in response to flooding stress

After 48 hours of flooding, 3 Sl*ARFs* (*SlARF2B*, *SlARF7A* and *SlARF9A*) were significantly downregulated and 4 were upregulated (*SlARF5*, *SlARF6B*, *SlARF17* and *SlARF24*) in leaves (Fig 3). The highest changes were observed within *SlARF7* and *SlARF17* whose expression was three times more repressed or ten times more induced respectively. As validation of the flooding stress, the expression of the *amino-cyclopropane -1- carboxylate oxidase* (*ACO1)* and *pyruvate decarboxylase 1* (*PDC1)* flooding marker genes was substantially increased in both leaves and roots as reported by Nie et *al*., (2002) [50] and Gharbi et *al*., (2007) [62], respectively. In root parts, *SlARF7A* was strongly repressed (512 times) (Fig 3).

Investigating the expression of SlARFs gene family in salt, drought and flooding conditions have shown that several members of these transcriptional factors are regulated. Among the candidate genes identified, *SlARF8A* and *SlARF10A* were significantly regulated in salt and water stresses. Moreover, for these two genes, we were able to generate transgenic lines expressing promoter-GUS construct (pARF8A::GUS or pARF10As::GUS). All this prompt us to check *SlARF8A* and *SlARF10A* genes expressions *in planta* in response to salt and drought stresses and also to examine a possible involvement of related miRNA in their regulation.

### Spatio-temporal analysis of SlARF8A and SlARF10A expression under salt and drought stress conditions

**Fig 4:**
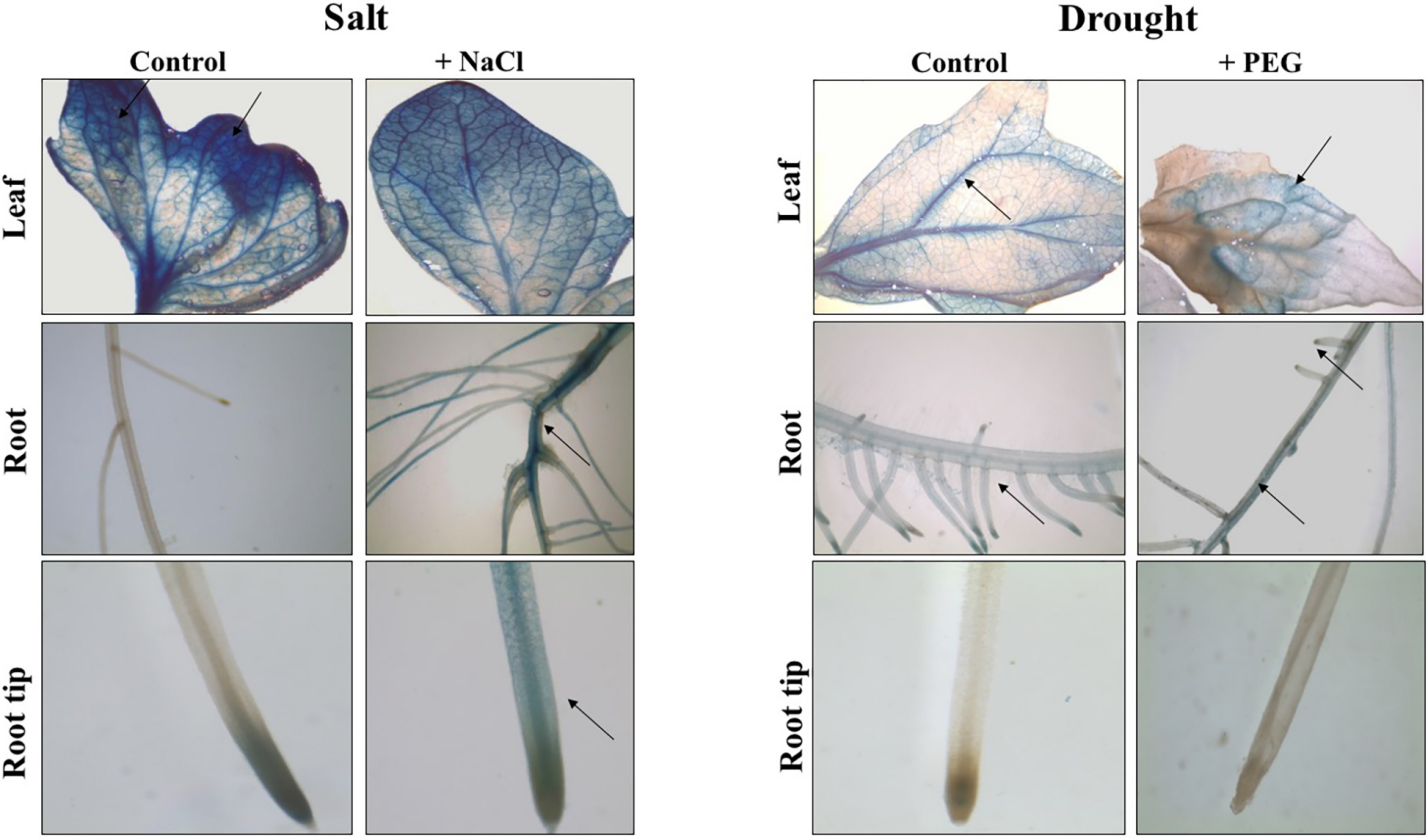
GUS activity in pARF8A::GUS tomato lines in salt or drought stress conditions. Salt and drought stresses were performed on three week-old tomato plants by adding 250 mM of NaCl or 15% PEG 20000 to the nutrient solution. Black arrows show the location of the GUS activity in the different tissues analyzed.

Since *SlARF8A* and *SlARF10A* were significantly regulated by salt and drought stresses, we sought to examine their spatiotemporal expression *in planta*. Tomato transgenic lines harboring pARF8A::GUS or pARF10A::GUS constructs were therefore generated and GUS expression was analyzed under stress conditions. GUS staining performed on three week-old plants expressing pARF8A::GUS fusion construct and exposed to 24 hours salt stress reveals a strong expression in leaves and lateral roots compared to untreated plants showing GUS expression only in leaves top and leaves veins (Fig 4). After 5 days of drought stress, GUS activity was detected in the primary root and in the middle part of leaves while the control plants showed a uniform blue coloration in leaf veins, primary root and lateral root tips (Fig 4).

**Fig 5:**
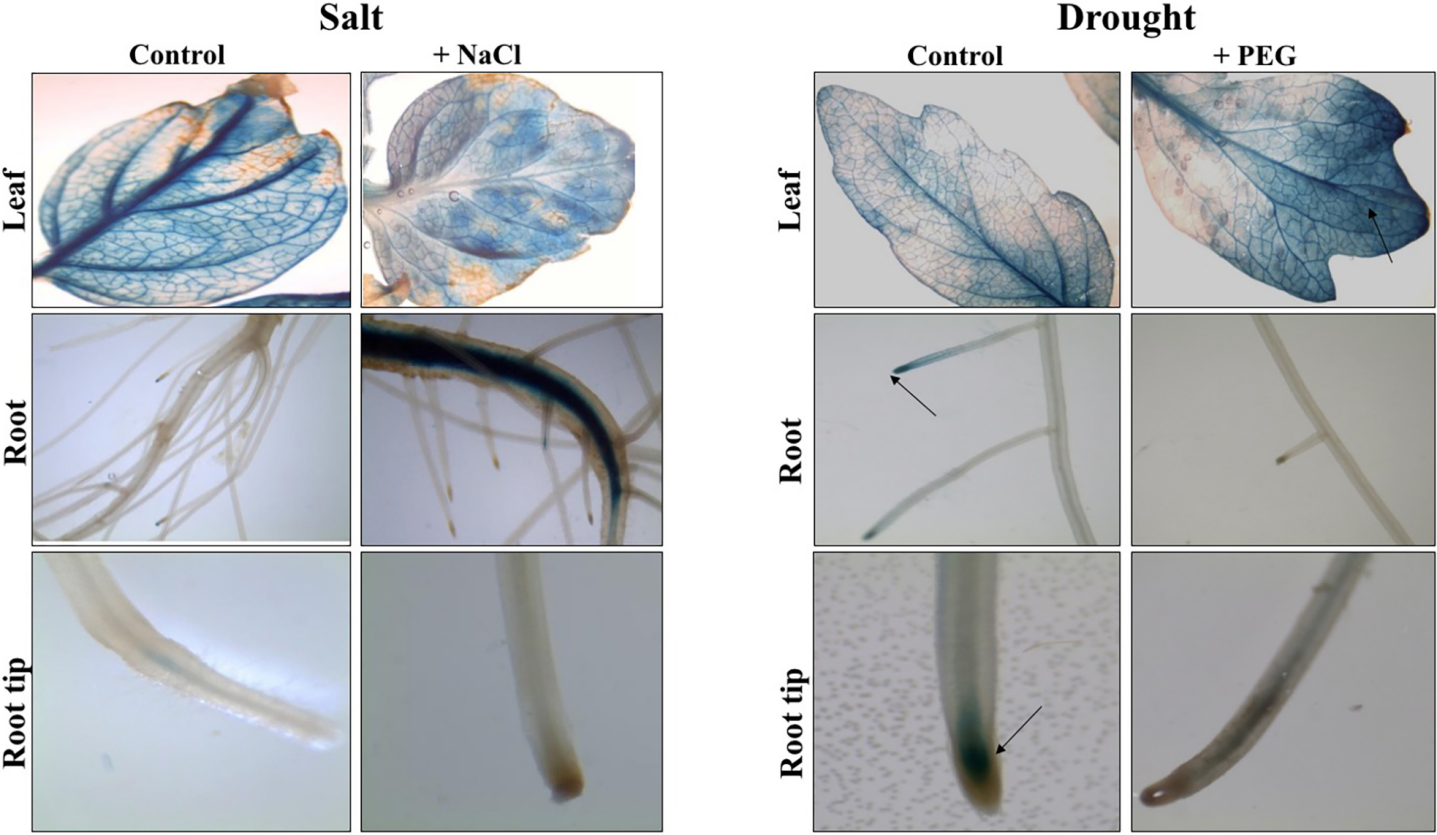
GUS activity in pARF10A::GUS tomato lines in salt or drought stress conditions. Salt and drought stresses were performed on three week-old tomato plants by adding 250 mM of NaCl or 15% PEG 20000 to the nutrient solution. Black arrows show the location of the GUS activity in the different tissues analyzed.

The GUS activity in pARF10A::GUS transgenic plants remained similar to the control plants after 24 hours on salt stress application in leaf tissues while it becomes intense in stressed roots. The control showed a uniform blue coloration all over the leaf. Five days exposure to drought restricted the GUS expression to the apical part of the leaf while the control plants displayed a blue coloration in the whole leaf and in the primary and lateral root tips (Fig 5).

### SlARF specific miRNAs responsiveness under abiotic stress conditions

**Fig 6:**
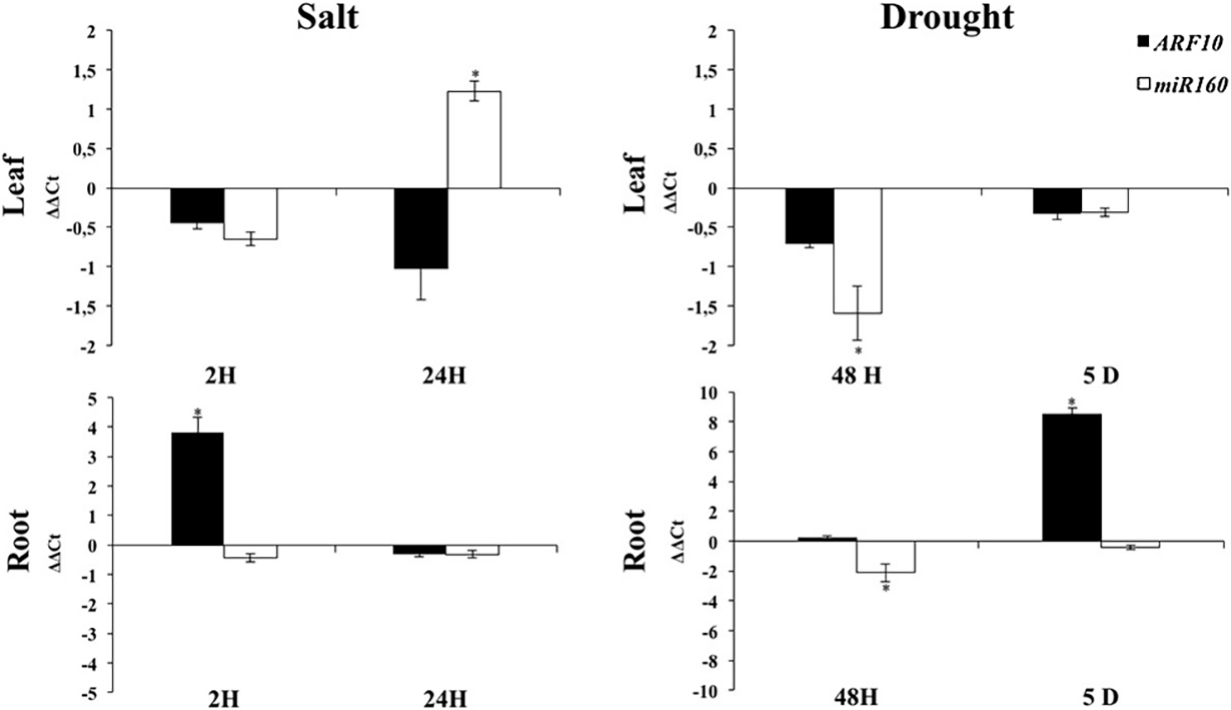
*SlARF10A* and *miR160* expression under salt and drought stress conditions. Values are mean ± SD of three biological replicates. Stars (*) indicate the statistical significance (p<0,05) using Student’s t-test.

miRNAs are small non-coding RNA molecules that negatively regulate their target genes at the post-transcriptional level [38,42]. ARF family members are known to be subject to this type of regulation. *SlARF8* and *SlARF6* are known to be targeted by *miR167* and *SlARF10* is specifically regulated by *miR160* [41,63]. Since the data described above (Fig 3) indicated that the expression of *SlARF8A* and *SlARF10A* was altered by both salt and drought stress, we checked whether the modification in the expression of these SlARF was correlated with the expression of their specific miRNA regulators. qPCR analysis showed that the expression pattern of *miR160* and its target gene *SlARF10A* changed significantly in response to salt stress (Fig 6). In leaves, *miR160* was two times induced after 24 hours of salt treatment while *ARF10A* gene was concomitantly downregulated. In roots, *SlARF10A* was up-regulated during the first two hours of salt treatment while the expression of *miR160* remained unchanged. Tomato *miR160* was significantly repressed in leaves after 48 hours of drought stress whereas the expression of *ARF10A* showed a high induction in 5 days drought stressed roots while the expression of *miR160* remained similar to the control.

**Fig 7:**
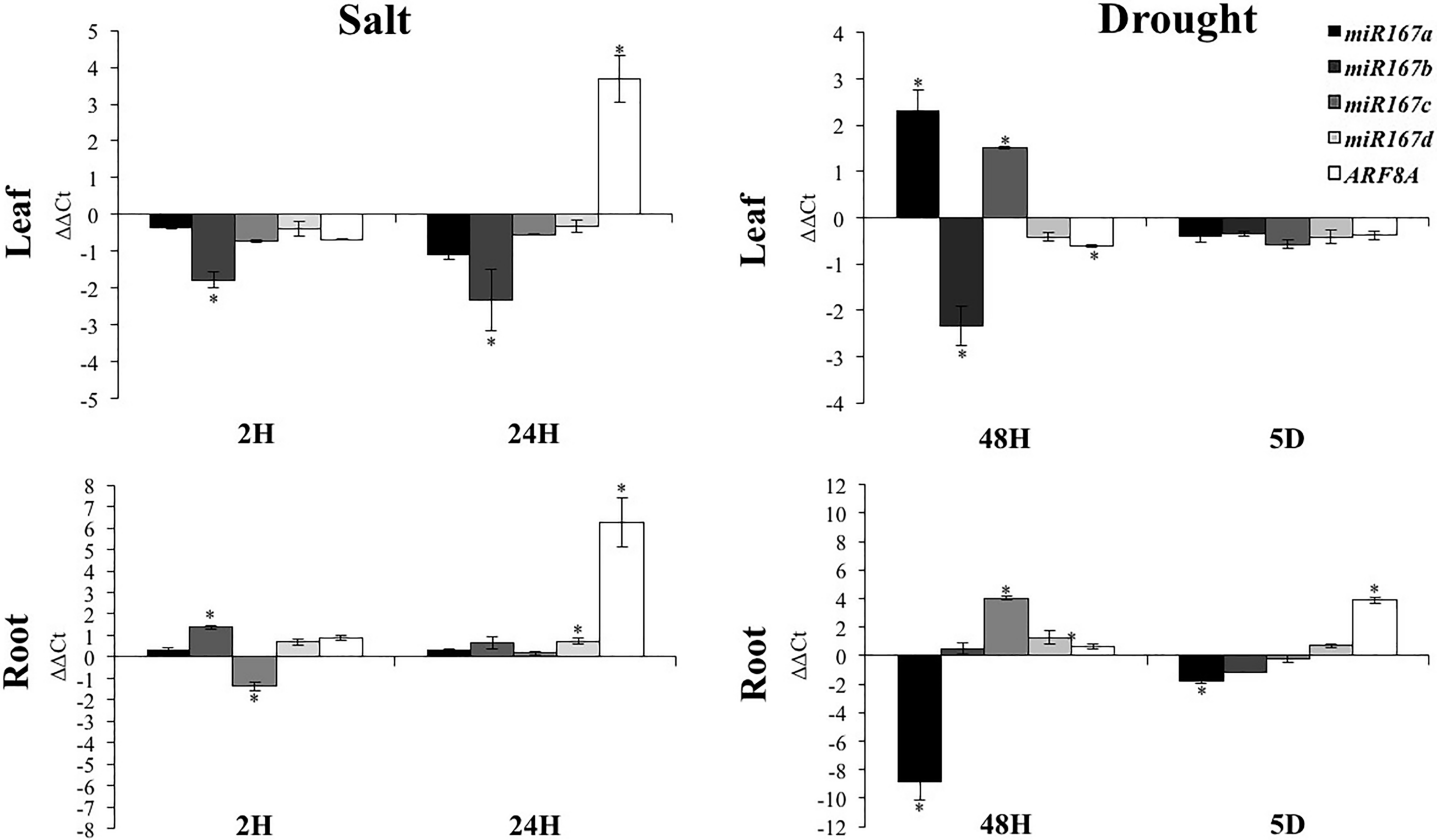
*SlARF8A* and *miR167s* (*miR167a*, *miR167b*, *miR167c*, *miR167d*) expression under salt and drought stress conditions. Values are mean ± SD of three biological replicates. Stars (*) indicate the statistical significance (p<0,05) using Student’s t-test.

The expression of miR167 precursors and their target gene *SlARF8A* were modified by salt and drought stresses (Fig 7). In salt stressed leaves, *SlARF8A* was highly induced up to 16 times after 24 hours of salt treatment while the expression *miR167b* was in a concurring of way highly repressed. In roots, *SlARF8A* was highly up-regulated after 24 hours of salt treatment while no strong changes occurred in the expression of *miR167* precursors. After 48 hours of drought stress exposure, *SlARF8A* gene expression in leaves was not strongly affected while *miR167a* and *miR167c* genes were highly induced when *miR167b* was repressed. In roots, *miR167a* was highly repressed when *miR167c* was induced while *SlARF8A* expression did not change. Five days of drought stress exposure resulted in an over-expression of *ARF8A* gene and down expression of *miR167a* in roots.

## Discussion

Plants are constantly exposed to various abiotic and biotic stresses in their immediate environment, limiting considerably their growth rate and productivity. Plants have evolved complex signaling pathways in response to various stimuli such as salt, drought, cold, wounding, or pathogen invasion in order to minimize damages while conserving valuable resources for growth and reproduction. These responses were found to be mediated by plant growth regulators [64]. Several phytohormones such as abscisic acid, ethylene, salicylic acid and jasmonate were identified as key regulators in various stress responses. Auxin is implicated in many plant developmental processes and recent studies suggest its involvement in stress [65,66]. It has been reported that the endogenous auxin level decreased substantially upon drought stress conditions but increased in response to cold and heat stresses in Rice and during pathogen infection in *Arabidopsis thaliana* [67–69]. The expression of *Aux⁄IAA* and *ARF* genes is altered during cold acclimation in *Arabidopsis thaliana* and during salt, water stresses and biotic stresses in *Oryza sativa* [16,70,71]. In this work, we investigated the implication of some actors of auxin signalling pathway in tomato responses to biotic and abiotic stresses.

### Auxin signaling pathway is altered by pathogen infection in tomato

The auxin response has emerged recently as an active actor in plant defense against pathogens [72]. Auxin coordinates plant development essentially through two transcriptional regulators Aux/IAA and ARFs [24]. In tomato, 22 *SlARFs* were previously isolated and characterized [21]. *In silico* promoter analysis of *SlARFs* using Plant PLACE database have revealed that at least one copy of the pathogenesis induced element (GT1GMSCAM4) was found in *SlARFs* 5’ regulatory regions. Furthermore, the expression of almost the entire *SlARF* gene family was altered in response to some of the pathogen tested based on the results related to biotic stresses that are available in TOMEXPRESS platform [59]. This suggests that auxin might be involved in biotic stress responses through ARFs. Other previous studies have underlined the involvement of auxin responsive genes in biotic stress responses. Auxin responsive genes are downregulated in *Arabidopsis thaliana* upon B*otrytis cinerea* infection [73]. In cotton, gene expression profiling in response to infection with *Fusarium oxysporum f*. *sp vasinfectum* also revealed differential expression of auxin responsive genes [74]. Studies conducted on rice had revealed that only two *OsARFs* were responsive to *Magnaporthe grisea* (ascomycete fungus) and *Striga hermonthica* (obligate root hemiparasite) infection [71]. The present results revealed that the expression of the *SlARF* gene family was altered by pathogen infections. Our findings suggest that the regulation of auxin pathway might be an important aspect of the defense response through the regulation of the expression of *ARF* gene family in tomato.

### Auxin accumulation is modified within high salinity and water deficit in tomato

Auxin and its gradients are found to be closely associated to some morphological changes observed in plants exposed to environmental stresses. These changes are likely associated with lateral root formation and axillary branching and represent one of the most specific responses to abiotic stresses [75,76]. In tomato, we showed that auxin distribution is modified by the stress application. Auxin was accumulated only in lateral root primordia and tips of DR5::GUS tomato transgenic plants exposed to salt and drought stress conditions. In support to these results, Zolla et *al*., (2009) [77] reported that salt stress stimulates the development of a larger number of lateral root primodia in *Arabidopsis*. This stimulation was associated with auxin accumulation in the lateral root primordia [78–80].

### Auxin response factors are potential key actors in abiotic stress responses in tomato

*In silico*, *SlARFs* promoter analysis has shown that *SlARF* regulatory regions were enriched with stress associated motifs. Drought and salt related *cis*-*acting* elements were found in all the 22 *SlARF* promoter regions. *SlARF1*, *SlARF4*, *SlARF8A*, *SlARF19* and *SlARF24* regulatory regions were more enriched in the salt induced element (GT1GMSCAM4). The promoter regions of *SlARF3* and *SlARF9A* although present respectively one copy of the GT1GMSCAM4 salt induced element. The MYC (MYCATERD1, MYCATRD22 and MYCCONSENSUSAT) were found in several copies *SlARF1*, *SlARF4*, *SlARF6A*, *SlARF7A*, *SlARF8A*, *SlARF9A* and *SlARF18* regulatory regions while the MYB motifs (MYBCORE, MYB1AT, MYB2AT and MYB2CONSENSUSAT) was highly present in *SlARF2A*, *SlARF2B*, *SlARF4*, *SlARF6B SlARF9A* and *SlARF18* promoter regions. All these finding suggest that the functions of these transcriptional factors may be associated with environmental stresses response. Moreover, investigating the expression of Auxin Response Factor family under abiotic stress conditions showed that most of the tomato ARFs were responsive and some of them were significantly regulated. Among the regulated genes, *SlARF1*, *SlARF4*, *SlARF8A*, *SlARF19* and *SlARF24* showed a significant upregulation in response to salt stress while *SlARF1*, *SlARF2A*, *SlARF2B SlARF4*, *SlARF6A*, *SlARF6B*, *SlARF7A*, *SlARF8A*, *SlARF9A* and *SlARF18* were substantially induced in drought stress conditions. In rice, several OsARFs were shown to be involved in salt and drought stress responses. Jain and Khurana (2009) [11] had reported that both *OsARF11* and *OsARF15* genes had shown differential expression in salt stress conditions and suggested that they are involved in rice response to stress. Zhou et *al*., (2007) [81] had shown the involvement of nine *ARFs* genes in response to water deficit (*OsARF2*, *OsARF4*, *OsARF10*, *OsARF14*, *OsARF16*, *OsARF18*, *OsARF19*, *OsARF22* and *OsARF23*). In *Sorghum bicolor*, Wang et *al*., (2010) [17] reported that *SbARF10*, *SbARF16*, and *SbARF21* genes were significantly induced in leaves and roots tissues exposed to drought conditions. However, among the 50 *ARF* isolated and identified in Soybean (*Glycine max*), only *GmARF33* and *GmARF50* were responsive (induced) to water deficit and were suggested as excellent candidates for drought stress responses in this plant [18].

The expression of some SlARFs showed different pattern in leaf and root under the same treatment. This difference might be associated with the function of these SlARFs. Some ARF functions are more related to the underground part more than to the aerial part. This is the case for *SlARF7A*, whose expression was significantly repressed or induced respectively in shoot and root parts under drought treatment. This gene seems to act in the underground part of the plant. This assumption is supported by Okushima et al., (2007) [82] and Goh et al., (2012) [83] who had shown that *ARF7* is involved in the control of lateral root formation in *Arabidopsis*.

Functional studies have shown that ARF genes are involved in the control of plant growth and development. Actually, Okushima et *al*., (2007) [82] and had reported that *AtARF7* acts synergistically with *AtARF19* in the control of lateral root formation and hypocotyl gravitotropism. Otherwise, *Solanum lycopersicum ARF2* has been proposed as a key regulator of apical hook formation while, in *Arabidopsis thaliana*, the same gene seems to act as a positive activator of flowering senescence and abscission and as a repressor of cell growth in the presence or absence of light and differential hypocotyl growth [84,85]. All these results suggest that auxin, through *ARF* gene family, activates stress specific auxin response genes in order to mitigate the negative effects of abiotic stresses.

### ARF8A and ARF10 are good indicators for salt and drought response in tomato

Our data have shown that *SlARF* gene family expression is modified by salinity and water deficit. Among these genes, *SlARF8A* and *SlARF10A* expressions are altered by the abiotic stress conditions. Moreover, their regulatory regions contain several copies of salt and/or drought induced elements. Investigating their expression *in planta* had revealed that their expression is clearly modified by salt and drought stress conditions. We found that *SlARF8A* promoter is especially expressed in lateral roots after 24 hours of exposure to both stresses. Previous studies confirmed the involvement of *ARF8* in abiotic stress response. Tian et *al*., (2004) [86] have suggested that *AtARF8* is stably expressed in lateral roots of *Arabidopsis thaliana* under light conditions and appears to control hypocotyl elongation.

Histochemical analysis of pARF10A::GUS tomato lines in salt stress conditions revealed that *ARF10A* gene is particularly more expressed in primary and lateral roots. The *SlARF10A* regulatory region presents four different salt/drought related motifs, which suggests that this gene might be implicated in root growth and development under stress conditions. Wang et *al*., 2005 [87] have found that *Arabidopsis thaliana ARF10A* is implicated in root formation and architecture by restricting cell division and promoting cell differentiation in the distal region which might suggest its implication in root development under stress conditions.

### miRNAs contribute to ARF gene regulation under stress conditions

miRNA are post transcriptional regulators of a large number of target genes by guiding target mRNAs for degradation or by repressing translation. Zhao and Srivastava (2007) [88] reported that some miRNAs are regulated and could be involved in cell responses to abiotic stresses such as salinity, cold and dehydration. Some miRNAs, including *miR160* and *miR167*, known to regulate the levels of transcription factor transcripts and protein abundance showed altered expression profiles in salt and drought conditions. Our results show that the downregulation of *miR167* precursors (*miR167a*, *miR167b*, *miR167c* and *miR167d*) was negatively correlated with the accumulation of *SlARF8A* transcripts in leaves after 24 hours of salt exposure. This finding was also observed in roots after 5 days of drought treatment. Knowing that *ARF8A* is implicated in control and development of vegetative and floral organs in Dicots, the downregulation of *miR167* gene precursors might enhance the auxin response and thus enhance shoot and leaf development. In cassava (*Manihot esculenta*), Xia et *al*., (2014) [89] showed that *miR167* expression was modified in response to extreme temperature and could induce the cleavage of its target gene, *ARF8*, due to the presence of the *miR167* cleavage sites on it. The expression of *miR160* was also negatively correlated with the expression of its target gene *SlARF10A* in roots exposed to drought stress. This finding suggests that *miR160* might be implicated in the post-transcriptional regulation of the expression of *ARF10A* gene under stress conditions.

## Conclusion

Auxin plays crucial roles in various aspects of plant growth and development. This phytohormone acts on the transcriptional regulation of target genes, mainly through Auxin Response Factors. The current study provide several clues on the potential involvement of many ARF genes as mediators of the auxin action in biotic and abiotic stress responses in tomato. The 5’ regulatory regions analysis of the *SlARFs* genes indicates the presence of several biotic and abiotic stress-responsive *cis*-elements. Moreover, transcriptome analysis reveals the responsiveness of *SlARF* genes to a wide range of biotic and abiotic stresses. Additionally, *SlARF8A* and *SlARF10A* specific *miRNA* were involved in the regulation of these genes suggesting the importance of post-transcriptional regulation in auxin signaling pathway and plant response to abiotic stresses. Taking together, the data presented in this work brings new elements and open new ways to explore the molecular mechanisms associated with stress tolerance in tomato. This provides new insights into tomato selection and breeding for environmental stress-tolerant.

## Supporting information

S1 Table Quantitative RT-PCR primers of SlARFs genes, stress marker genes and miRNA gene precursors. (PDF)

S2 Table SlARFs RNA levels in tomato leaves in the control plants and upon pathogen infections. The data presented in this table were extracted from TOMEXPRESS database [44].
(PDF)

S3 Table Potential Cis-acting regulatory elements identified in the 5’ regulatory sequences of tomato Auxin response factor gene family. The 2 Kb of 5’ regulatory region were analyzed using the Place software.
(PDF)

S1 Fig Map of the 5’ regulatory sequences of *Solanum lycopersicum* auxin response factors gene family. The consensus sequences corresponding to the various putative cis-elements are described in S2 Table. Positions are with respect to the first base of translation start site.
(PDF)
