# Supplementary material for: Auxin Response Factors (ARFs) are potential mediators of auxin action in tomato response to biotic and abiotic stress (Solanum lycopersicum)
Source: PLoS One. 2018 Feb 28;13(2):e0193517. doi: 10.1371/journal.pone.0193517 (PMC5831009; doi:10.1371/journal.pone.0193517)
Supplement: S1 Table — (PDF) [file pone.0193517.s001.pdf]

Supplementary Material

Auxin Response Factors (ARFs) are potential mediators of auxin action in Tomato response to biotic and abiotic stress (*Solanum lycopersicum* L.)

Sarah Bouzroud[1,3], Sandra Gouiaa[1], Nan Hu[1], Anne Bernadac[1], Isabelle Mila[2], Najib Bendaou[3], AbdelAziz Smouni[3], Mondher Bouzayen[1], Mohamed Zouine[1*]

***Correspondence:** Mohamed ZOUINE: mohamed.zouine@ensat.fr

Supplementary Table S1: Quantitative RT-PCR primers of *SlARF* genes, stress marker genes and the miRNA gene precursors

Gene name	Forward primer sequence	Reverse primer sequence
SlARF1	TCTCCTTCATCATTCTCATACTG	GAACCATTCTCACCATAACC
SlARF2A	GCAAGGTCAAGAGTTATCGA	CATTGGTTTCTCAGACAAGTC
SlARF2B	CACTTAATCCACTTCCAATACC	TACAACTACTTTGGATGAACCT
SlARF3	AATTGCAGTATCAGACTTTGG	TCTAGATATCCCAGAACTAGGA
SlARF4	CATTATTGTTGGTGACTTTGTG	GACCTTTGGAAACCTATTGG
SlARF5	CCTTCAGAGTTTGTCATTCCT	AACATCATTCCAAATCTCATACC
SlARF6A	CCAACATATCCCTAGTACTTCAG	GTGCCTGAGATATTAGTTGGT
SlARF6B	ACCCTCTAGTATCTTCATCCT	TCCGAGACCTTTGTATTGTG
SlARF7A	TCAACTCCTCAAACATACCT	TGAACTATCCAAATAATCCATCTG
SlARF8A	TGACATCGAATGGAAATTCAG	GTCTCTTAGCACTAACAAACAC
SlARF8B	GTCAGTCCGTGATCATAGAG	GGAATCCAAGCTACAATTTCC
SlARF9A	ATCATTCAATCTCAAATCAAAGGT	CCTCATCATTGTCTTCTTCAG
SlARF10A	ATTCTCTGTGCCTAGATACTG	CTATAAATGTGCCTAAACTTCCA
SlARF17	TGAAGTTGATGAAGTTACTATGAG	TCCTCCATTATTCGCATCTG
SlARF18	AATCTACACTCGGCATTGTC	AAGCTTCCTATCTTATCATTGGA
SlARF19	TGGTGGATGAATCTGTTGTC	TACTTAGACAGCTCTGAACCT
SlARF24	TCATTGTTGGATGTTTCAAAGG	GAAGTCTTGGAAAGTAGTATACTC
Actin	TGTCCCTATCTACGAGGGTTATGC	AGTTAAATCACGACCAGCAAGAT
CI17	GGCAATTTCATCTGAGTTGTCTGA	CTATTTGATCGATGAAGTTTCTTTTCC
PDC1	TCTTTGAGCACATTCAGAGG	AACAGACCAACCAATAGATCCA
ACO1	AAGGGACTCCGCGCTCA	AGTTGAAGGCCACTCACTTTGT
**miR160**	CCTGGATGCCAACTAAGAACAG	GGTACCAACTAGGAAGAGTGTC
**miR167a**	TCCGAGCATGACCTAACCTT	TCAAAACCCTACATCCCAAA
**miR167b**	CAATTCTGAACCCTGGCCTA	GACAGGTAAGGCTGCCACAT
**miR167c**	TGGATCGTGAACCACTAGCA	GCACAATTAGACCCTTGTTGC
**miR167d**	GCCACATGATCTGATCTTTCCTC	GCCAGCATGATCTAAACTTTCC
