# Supplementary material for: Auxin Response Factors (ARFs) are potential mediators of auxin action in tomato response to biotic and abiotic stress (Solanum lycopersicum)
Source: PLoS One. 2018 Feb 28;13(2):e0193517. doi: 10.1371/journal.pone.0193517 (PMC5831009; doi:10.1371/journal.pone.0193517)
Supplement: S2 Table — The data presented in this table were extracted from TOMEXPRESS database [44]. (PDF) [file pone.0193517.s002.pdf]

Supplementary Material

Auxin Response Factors (ARFs) are potential mediators of auxin action in Tomato response to biotic and abiotic stress (*Solanum lycopersicum* L.)

Sarah Bouzroud[1,3], Sandra Gouiaa[1], Nan Hu[1], Anne Bernadac[1], Isabelle Mila[2], Najib Bendaou[3], AbdelAziz Smouni[3], Mondher Bouzayen[1], Mohamed Zouine[1*]

***Correspondence:** Mohamed ZOUINE: mohamed.zouine@ensat.fr

Supplementary Table S2: *SlARFs* RNA levels in tomato leaves in the control plants and upon pathogen infections. The Data presented in this table were extracted from TOMEXPRESS database.

	RIO GRANDE tomato control plants	RIO GRANDE tomato leaves exposed to *P.synrigae*	RIO GRANDE tomato leaves exposed to *P.putida*	RIO GRANDE tomato leaves exposed to *P.fluorescens*	RIO GRANDE tomato leaves exposed to *A.tumefasciens*	RIO GRANDE tomato leaves exposed to Flagellin	CLN2777A tomato control plants	CLN2777A tomato exposed to *YCV*
Sl-ARF1	0,2732	0,2394	0,3743	0,3076	0,274	0,3095	0,4074	0,295
Sl-ARF2A	0,5404	0,3959	0,3208	0,3575	0,6185	0,3031	0,3766	0,3227
Sl-ARF2B	0,1982	0,1385	0,1336	0,1375	0,1867	0,143	0,1075	0,0988
Sl-ARF3	0,1208	0,1258	0,1143	0,1104	0,1284	0,1063	0,0823	0,103
Sl-ARF4	0,1415	0,09	0,0529	0,0728	0,1772	0,0444	0,3429	0,1845
Sl-ARF5	0,0097	0,0102	0,0056	0,0061	0,0107	0,0043	0,136	0,0474
Sl-ARF6A	0,0587	0,0505	0,0548	0,0669	0,0508	0,0789	0,0469	0,0288
Sl-ARF6B	0,1641	0,1247	0,1364	0,1546	0,1478	0,1322	0,0363	0,0394
Sl-ARF7A	0,112	0,086	0,0704	0,0855	0,1127	0,0776	0,083	0,0823
Sl-ARF7B	0,1164	0,0937	0,0671	0,0942	0,116	0,0893	0,0509	0,0673
Sl-ARF8A	0,3882	0,5074	0,2481	0,2766	0,3797	0,2815	0,2728	0,254
Sl-ARF8B	0,0539	0,0615	0,0372	0,0439	0,0518	0,0339	0,2788	0,1072
Sl-ARF9A	0,0028	0,0025	0,0019	0,0019	0,0031	0,0011	0,0551	0,0236
Sl-ARF9B	0,0499	0,0373	0,0281	0,0334	0,0427	0,0249	0,0314	0,0245
Sl-ARF10A	0,0884	0,0588	0,071	0,0865	0,0915	0,0626	0,0497	0,0516
Sl-ARF10B	0,0142	0,0071	0,0074	0,0087	0,011	0,0072	0,0103	0,0123
Sl-ARF16A	0,066	0,0418	0,0339	0,0489	0,0594	0,0361	0,1024	0,0797
Sl-ARF16B	0	0	0	0	0	0	0	0
Sl-ARF17	0,0171	0,0076	0,0081	0,0111	0,0221	0,0072	0,0167	0,0149
Sl-ARF18	0,1557	0,1865	0,1854	0,1524	0,1445	0,248	0,4036	0,3645
Sl-ARF19	0,1007	0,0759	0,0636	0,0722	0,0835	0,0865	0,054	0,0697
Sl-ARF24	0,0712	0,0786	0,0649	0,0725	0,0624	0,0592	0,0487	0,0385
