# Supplementary material for: Auxin Response Factors (ARFs) are potential mediators of auxin action in tomato response to biotic and abiotic stress (Solanum lycopersicum)
Source: PLoS One. 2018 Feb 28;13(2):e0193517. doi: 10.1371/journal.pone.0193517 (PMC5831009; doi:10.1371/journal.pone.0193517)
Supplement: S3 Table — The 2 Kb of 5’ regulatory region were analyzed using the Place software. (PDF) [file pone.0193517.s003.pdf]

Supplementary Material

Auxin Response Factors (ARFs) are potential mediators of auxin action in Tomato response to biotic and abiotic stress (*Solanum lycopersicum* L.)

Sarah Bouzroud[1,3], Sandra Gouiaa[1], Nan Hu[1], Anne Bernadac[1], Isabelle Mila[2], Najib Bendaou[3], AbdelAziz Smouni[3], Mondher Bouzayen[1], Mohamed Zouine[1*]

***Correspondence:** Mohamed ZOUINE: mohamed.zouine@ensat.fr

Supplementary Table S3: C*is*-acting regulatory elements identified in the 5’ regulatory sequences of tomato Auxin response factors gene family. The 2kb of 5’ regulatory region was analyzed using the Place software.

Cis-acting element	Sequence	Function	Reference
WBOXNTCHN48	CTGACY	« W box » Elicitor-responsive	(Deb and Kundu 2015)
WBOXTERF3	TGACY	« W box », response to wounding	(Nishiuchi et al. 2004)
RAV1AAT	CAACA	RAV1 binding site, cold responsiveness	(Guy 1999)
MYB1AT	WAACAA	Element involved in dehydration responsiveness	(Abe et al. 2003)
MYBCORE	CNGTTR	Element involved in water stress response	(Abe et al. 2003)
GT1GMSCAM4	GAAAAA	Pathogenesis and salt-induced element	(Trivedi et al. 2013)
CCAATBOX1	CCAAT	HSE (Heat Shock Element)	(Sohn et al. 2006)
MYB2AT	TAACTG	Element involved in response to water stress and ABA	(Abe et al. 2003)
MYB2CONSENSUSAT	YAACKG	MYB recognition site involved in dehydration and ABA response	(Abe et al. 2003)
MYCATERD1	CATGTG	MYC binding site involved in response to dehydration and ABA	(Abe et al. 2003)
MYCATRD22	CACATG	MYC binding site involved in response to dehydration and ABA	(Abe et al. 1997)
MYCCONSENSUSAT	CANNTG	Cis-acting regulatory element involved in early response to drought and ABA induction	(Dang et al. 2011)
