# Supplementary material for: Auxin Response Factors (ARFs) are potential mediators of auxin action in tomato response to biotic and abiotic stress (Solanum lycopersicum)
Source: PLoS One. 2018 Feb 28;13(2):e0193517. doi: 10.1371/journal.pone.0193517 (PMC5831009; doi:10.1371/journal.pone.0193517)
Supplement: S1 Fig — The consensus sequences corresponding to the various putative cis-elements are described in S2 Table. Positions are with respect to the first base of translation start site. (PDF) [file pone.0193517.s004.pdf]

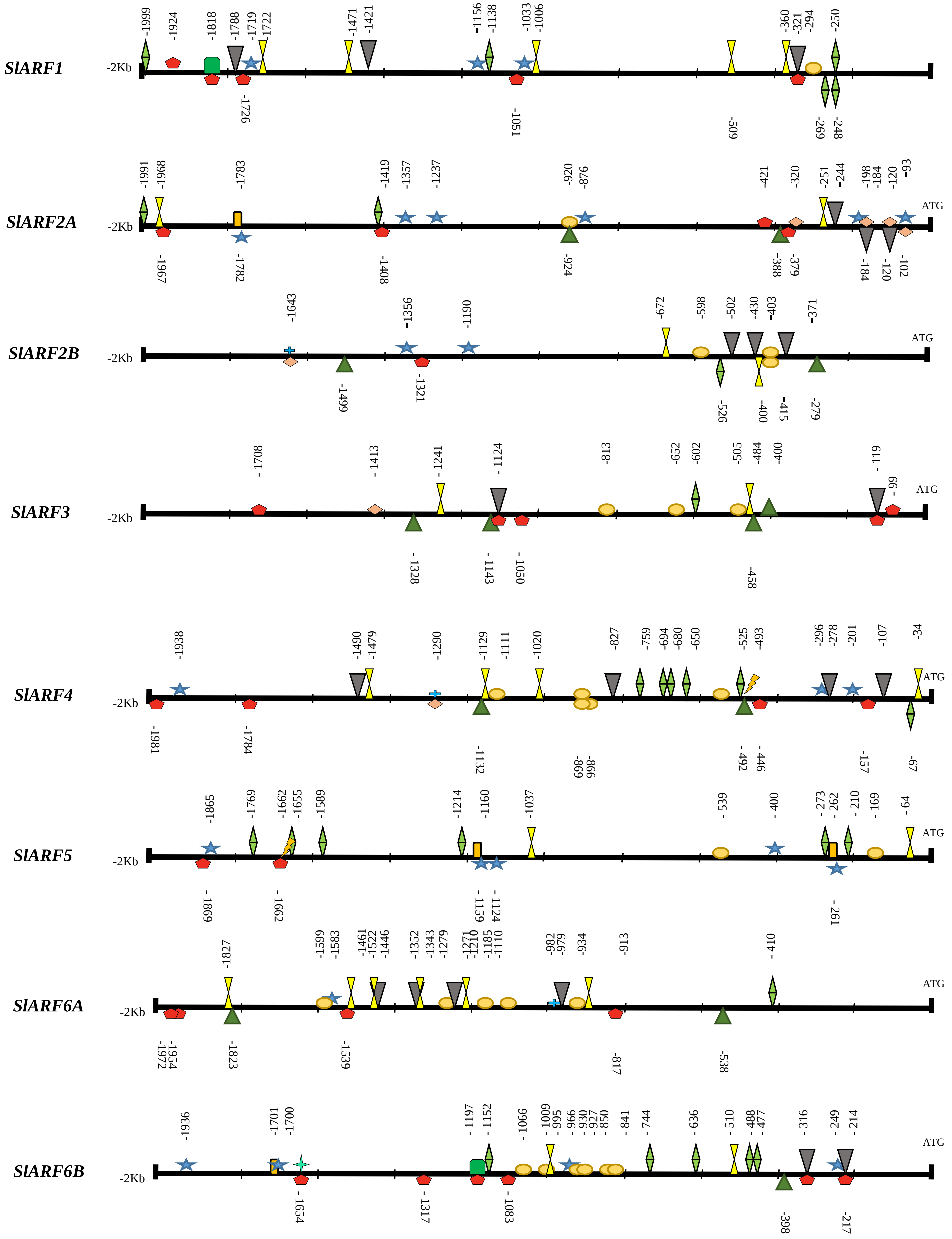
SlARF1
SlARF2A
SlARF2B
SlARF3
SlARF4
SlARF5
SlARF6A
SlARF6B
-2Kb
ATG

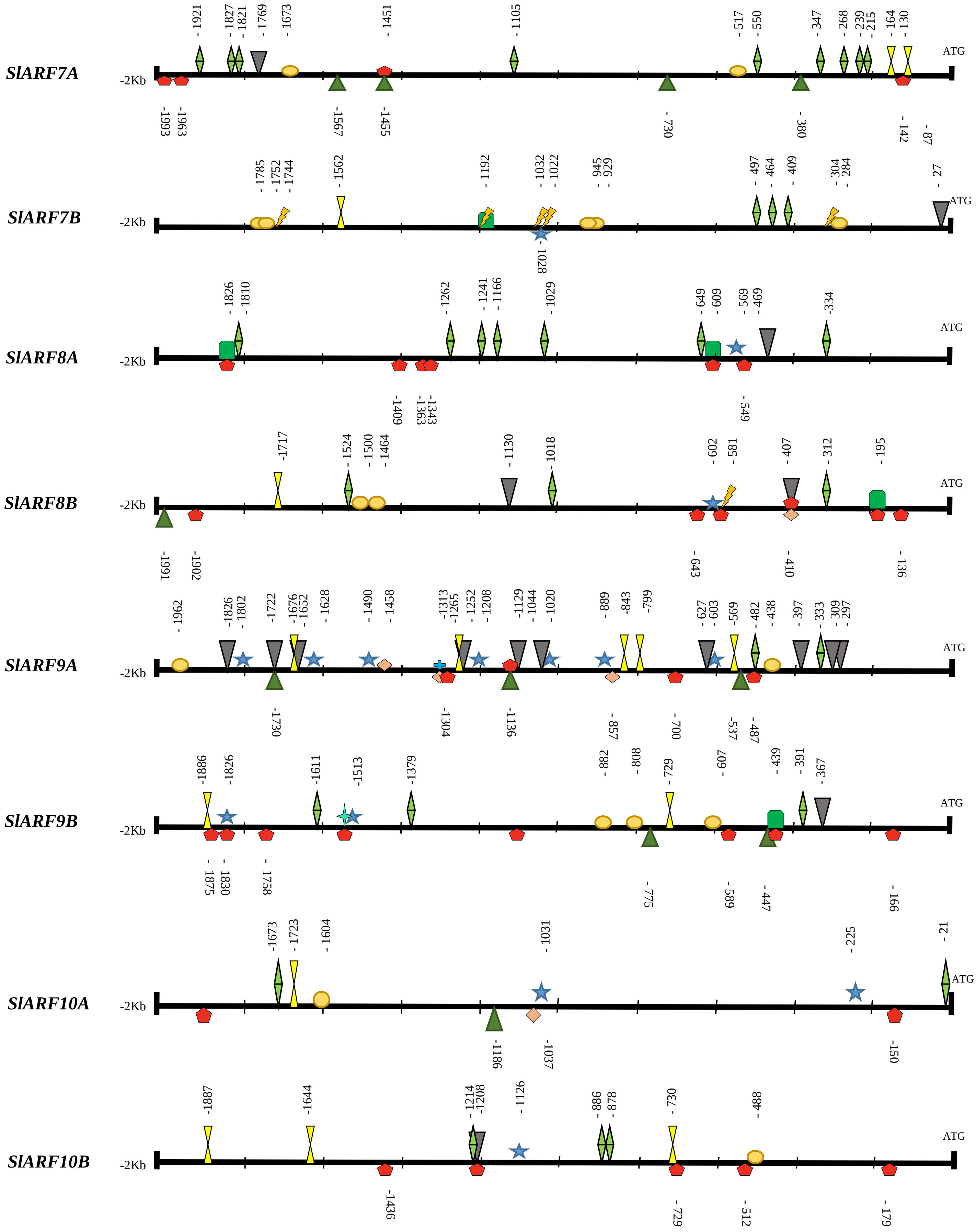

SlARF7A
-2Kb
ATG
- 1921 - 1827 - 1821 - 1769 - 1673 - 1451 - 1105 - 517 - 550 - 347 - 268 - 239 - 215 - 164 - 130
- 1993 - 1963 -1567 -1455 - 730 - 380 - 142 - 87
SlARF7B
-2Kb
ATG
- 1785 - 1752 - 1744 - 1562 - 1192 - 1032 - 1022 - 945 - 929 - 497 - 464 - 409 - 304 - 284 - 27
- 1028
SlARF8A
-2Kb
ATG
- 1826 - 1810 - 1262 - 1241 - 1166 - 1029 - 649 - 609 - 569 - 469 -334
-1409 -1363 -1343 - 549
SlARF8B
-2Kb
ATG
-1717 - 1524 - 1500 - 1464 - 1130 - 1018 - 602 - 581 - 407 - 312 - 195
-1991 -1902 - 643 - 410 - 136
SlARF9A
-2Kb
ATG
- 1962 -1826 - 1802 -1722 -1676 -1652 - 1628 - 1490 - 1458 -1313 -1265 - 1252 - 1208 -1129 -1044 - 1020 - 889 -843 -799 - 627 - 603 -569 - 482 - 438 - 397 - 333 - 309 - 297
-1730 -1304 -1136 - 857 -700 -537 -487
SlARF9B
-2Kb
ATG
-1886 -1826 -1611 -1513 -1379 - 882 - 808 - 729 - 609 - 439 - 391 - 367
- 1875 - 1830 - 1758 - 775 - 589 - 447 - 166
SlARF10A
-2Kb
ATG
-1673 - 1723 - 1604 - 1031 - 225 - 21
-1186 -1037 -150
SlARF10B
-2Kb
ATG
- 1887 -1644 - 1214 -1208 - 1126 - 886 - 878 - 730 - 488
-1436 - 729 - 512 - 179

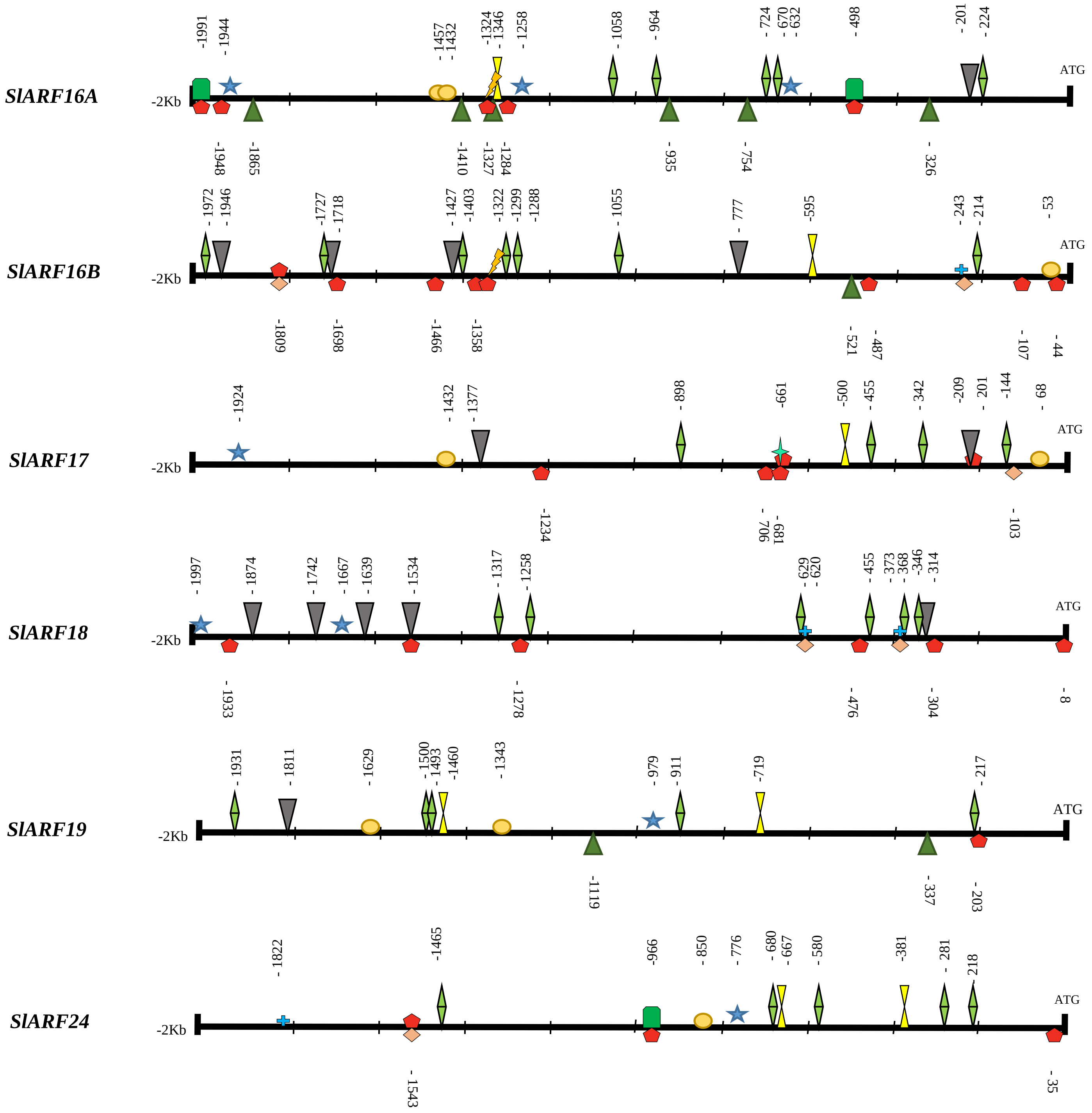

Supplementary Figure S1: Map of the 5’ regulatory sequences of *Solanum lycopersicum* auxin response factors gene family. The consensus sequences corresponding to the various putative cis-elements are described in Supplementary Table S3. Positions are with respect to the first base of the translation start site. Symbols legend:

WBOXNTCHN48; WBOXTERF3; RAV1AAT; MYCCONSENSUSAT; MYBCORE;
GT1GMSCAM4; CCAATBOX1; MYB2AT; MYB2CONSENSUSAT; MYCATERD1;
MYCATRD22; MYB1AT.
